# Evaluation of Q angle in differents static postures 

**DOI:** 10.1590/1413-78522014220600451

**Published:** 2014

**Authors:** Hugo Machado Sanchez, Eliane Gouveia de Morais Sanchez, Mario Antonio Baraúna, Roberto Sérgio de Tavares Canto

**Affiliations:** 1. Universidade de Rio Verde, Rio Verde, GO, Brazil, Universidade de Rio Verde (UniRV), Rio Verde, GO, Brazil; 2. Centro Universitário do Triângulo, Uberlândia, MG, Brazil, Centro Universitário do Triângulo (UNITRI), Uberlândia, MG, Brazil; 3. Universidade Federal de Uberlândia, Uberlândia, MG, Brazil, Universidade Federal de Uberlândia (UFU), Uberlândia, MG, Brazil

**Keywords:** Posture, Evaluation, Knee, Photogrammetry

## Abstract

**OBJECTIVE:**

To compare the value of Q angle in different positions, in the external and internal rotations of lower limbs.

**METHODS:**

This is a descriptive cross-sectional study. We have evaluated 62 volunteers, 32 women and 30 men in the following positions: supine positions with parallel feet, supine with abduction (external rotation of lower limbs), and standing position with parallel feet and with external rotation. All the participants were sedentary and without previous history of acute injury or complaints regarding lower limbs. In order to calculate the Q angle we used computerized biophotogrammetry through ALC image 2.1^(r)^ program.

**RESULTS:**

The results of the comparisons showed significant difference between the standing position with feet parallel and orthostatic positions with abductees feet on the left side for both genders (p = 0.000). We also found a significant difference between supine and standing position with abducted feet and with feet parallel on the left side (p = 0.046) in females.

**CONCLUSION:**

From these results, we can conclude that there are significant differences in the standing position with abducted feet and parallel to the left leg, and symmetry between the lower limbs independent of rotation of limbs in the supine posture. **Level of Evidence II, Diagnostic Studies Investigating a Diagnostic Test.**

## INTRODUCTION

The quadriceps angle, or Q angle, is the angle formed by the encounter of two lines, one that starts at the anterior iliac spine (AIS) and goes to the center of the patella, and another that goes from the tibial tuberosity to the center of the patella.^1^^-^ 7 It is a clinical measurement used to measure knee alignment with respect to the hip, femur and tibia, and to evaluate the patella alignment.^5^^,^ 8 ^,^ 9 However, there are not universally accepted normal or abnormal values of the Q angle due to lack of a coefficient of reliability of different methods and measures for this angle.^7^

According to Livingston and Mandingo,^8^ the symmetry of the Q angle between members can be the cause of disagreements, and because of that, in their study they evaluated 75 volunteers divided into three groups (no anterior knee pain, unilateral pain and bilateral pain) and found through goniometric analysis with volunteers in standing position, that the angle values were asymmetric for the three groups, but that this asymmetry varied between groups.

The importance of assessing the Q angle is cited by several studies involving biomechanics, clinics and knee surgery. This angle helps indicating the force vector acting on the patella and patellofemoral joint,^1^^,^ 2 ^,^ 5 ^,^ 6 ^,^ 9 ^-^ 11 furthermore, it is also used as a criterion to identify candidates for surgery or as predictor of risk of injury.^5^^,^ 6 ^,^ 9

Regarding the clinical significance of Q angle, it is observed that changes on this angle are associated with chondromalacia patella, lateral dislocation of the patella, erosion of the patellar cartilage and of the lateral condyle, femoral internal rotation, foot pronation and internal tibial torsion.^3^^,^ 4 ^,^ 7 According to Devan *et a*l.,^10^ changes in the Q angle in valgus knees alter the biomechanics and impairs muscle levers and, consequently, its functions.

The Q angle shows an inverse relationship with quadriceps strength, as the smallest the angle the greater the force produced by the quadriceps, which assumes that individuals with above normal Q angle have lower quadriceps strength and are more subject to diseases of the patellofemoral joint.^6^

The value of Q angle varies according to the patients gender, the state of contraction of the quadriceps and the position adopted by the patient, standing or lying down.^3^ The rotation of the lower limbs have direct influence on the alignment of the knees, altering them according to their positioning.^4^^,^ 5 ^,^ 8 ^,^ 14 ^-^ 16 The lack of a consensus on normal Q angle values is partly due to the absence of a standardized procedure of measuring the angle.^5^

Computed biophotogrammetry uses the photogrammetric principle to photographic images. It shows as a non-invasive resource, offering an obtaining and photointerpretation low cost system, as well as high precision and reproducibility. The image interpretation consists in constant observation of the image to completion and issuance of a relevant report. References are marked in individuals evaluated by computerized biophotogrammetry. This demarcation is fundamental to the study and analysis of data.^11^ Other studies have already used this resource to evaluate the Q angle, confirming its practicality and effectiveness.^7^^,^ 18

Because of the great clinical and biomechanical importance of evaluation of the Q angle, the aim of this study was to compare values ​​of Q angle in relation to supine and upright postures, including the default measurement position (neutral rotation position of the lower limbs with parallel feet) and the position with external rotation of the lower limbs.

## METHODS

This is a descriptive cross-sectional study. We randomly selected 62 volunteers - 32 men and 30 women, students at *Centro Universitário do Triângulo* (UNITRI) Uberlândia, MG, Brazil.

In this study were included sedentary university students without pathological complaints in the lower limbs. There were excluded individuals with musculoskeletal injuries in the lower limbs, lower limbs discrepancy greater than 1.5 cm, which have suffered fracture, dislocation, or previous surgery of the lower limbs, obese subjects, with diseases of bone, muscle or connective tissue, with neurologic pathologies or poliomyelitis sequelae and volunteers who were pregnant.

This study was performed upon approval of the Ethics Committee on Human Research of *Centro Universitário do Triângulo* - UNITRI (authorization No. 16). Participants were informed about the objectives of the study and were free to choose to participate or not in the study.

 The volunteers who agreed to participate in this study signed the Informed Consent Form and underwent an evaluation always performed by the same person. Then, the completion of the evaluation chart was made, that contained the personal data of the volunteers, the length of the lower limbs and issues related to the exclusion criteria. After this procedure, volunteers proceeded to the identification of anatomical landmarks and placement of markers, image capture, and photographic record.

Then, the volunteers remained barefoot and with appropriate clothing (shorts or underwear), so as the anatomical points be easily located and viewed. We chose to demarcate the bony prominences (tibial tuberosity and the anterior superior iliac spine) using palpatory anatomy, since the evaluators were trained and experienced, besides the fact that this is the method used by clinicians during routine assessments and has no radioactive effects, as in the case of demarcation using a radiological image. The point of the center of the patella was located through calipers to determine this point with reliability.

After identification of anatomical points, they were marked with self-adhesive circular label markers.

**Figure 1:**
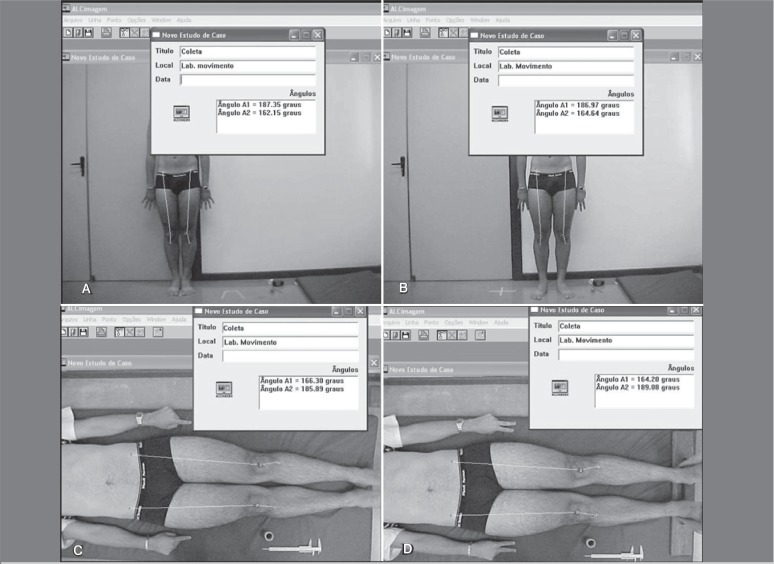
Positioning of volunteer for picture taking. A) Volunteer in orthostatism and parallel feet; B)Volunteer in orthostatism and abducted feet; C) Volunteer lying with parallel feet; D)Volunteer lying and abducted feet.

Once demarcated the anatomical points regarding the Q angle the process of data collection was carried out as described below. For standardization of the image, with the volunteer standing upright, two sites were previously demarcated on the ground, the first being demarcated following Livingston and Spaulding,^5^ according to which the feet are placed together touching medially (orthostatism with parallel feet - OPF), lined with an adhesive tape on the floor. The volunteers were instructed to keep the quadriceps muscle of both limbs relaxed, eyes to the horizon and arms along the body. (Figure 1A) Right after, we proceeded to obtain the second image, in which the volunteer remained in the standing position and kept a separation of 7.5 cm of the heels and an external rotation of forefoot of 10 degrees from the medium line^13^ (orthostatism with abductees feet - OAF), quadriceps muscles relaxed, eyes to the horizon and arms along the body. (Figure 1B) In order to obtain the images with the volunteers in the upright position, the camera was positioned on a tripod on level and in plumb at a 0.90m height and a distance of 2.90m from the volunteer, in order to capture the image of the hip to feet when standing up.

After collecting the second image, the volunteer was placed in the supine posture, anatomical points remarked and he was instructed to maintain the position without rotation of the lower limbs, i.e., keeping the hip in a neutral position (lying with parallel feet - LPF), knees extended, relaxed ankle, keeping therefore, his feet parallel and relaxed quadriceps muscles. (Figure 1C) For the acquisition of the fourth image, only the positioning of the feet was changed (lying with abducted feet - LAF), which were adjusted in the same way as that of the second image in orthostatism.^9^ (Figure 1D) For this position, a positioner was made that kept the legs of the volunteers in the desired external rotation. For this image acquisition with the volunteer in supine position, the camera was positioned on a support lateral to the mat, parallel to the ground at a 1.70m height in order to capture the image from the hip to the foot of each volunteer. The alignment of the lower limb of the volunteers was controlled by means of the positioning/rotating member, with no other adjustments concerning the joints of the foot or hip.^5^

The whole process of marking of anatomical landmarks and image acquisition was performed three times in each of the four positions on the above sequence, totaling 12 marks/images per volunteer, so we obtained three angular values for each position. To perform the statistical analysis we used the arithmetic average of the three values obtained for each position. The importance of remarking the points between each picture taking in the analyzed positions was because of the possible change of location of the adhesives markers when limbs were rolled members and/or when the posture of volunteers were modified.^9^

When the registration of these images was ended, they were transferred to a computer where the analysis using computed biophotogrammetry through the application ALC image 2.1^(r)^ was performed. This method have been validated and is highly reliable for angular calculation.^7^^,^ 18 The angular measurements were verified from union of anatomical points previously marked. In order to calculate the Q angle a line that started at the anterior superior iliac spine to the center of the patella and thence to the anterior tibial tuberosity was traced, drawing, in this way, an angle corresponding to the quadriceps angle.

In order to check the difference between the values of the Q angle between the limbs in the evaluated positions, we used the Student t test, as well as the comparison between the values with and without rotation of the lower limb. To investigate possible differences in Q angle on cross-comparison of the four positions of each limb the analysis of variance (ANOVA) followed by Tukey test were used. The level of significance was set at p less than 5%.

## RESULTS

The mean age of male subjects was 21.33 ± 2.20 years old, and of female subjects 20.81 ± 3.13 years old.

In Table 1 are shown the descriptive analysis of the data for men and women bilaterally. The four positions analyzed were lying parallel feet (LPF), lying abducted feet (LAF), orthostatic abducted feet (OAF) and orthostatic parallel feet (OPF).

**Table 1:** Descriptive analysis of data on the analyzed positions for men and women.

	Side	M	MD	St. Dev		Side	M	MD	St. Dev
**Men**	****	****	****	****	**Women**				
LPF	Left	7.85	8.29	6.51	LPF	Left	16.44	15	6.46
	Right	6.81	6.42	7.63		Right	14.4	13.7	6.35
LAF	Left	8.24	8.42	6.47	LAF	Left	15.43	15.43	6.23
	Right	7.09	7.62	7.80		Right	14.14	13.19	6.22
OAF	Left	7.34	7.97	7.84	OAF	Left	15.66	17.07	7.01
	Right	8.94	10.35	8.94		Right	17.71	19.21	7.2
OPF	Left	11.24	11.43	7.77	OPF	Left	19.79	19.21	7.16
	Right	10.13	10.21	7.40		Right	17.78	18.77	7.56
Mean		8.46	8.84	7.45	Mean		16.42	16.45	6.77

LPF: lying parallel feet; LAF: lying abducted feet; OAF: orthostatic abducted feet; OPF: orthostatic parallel feet; M: Mean; MD: median; St. Dev: Standard deviation.

The differences in values between right and left Q angle were calculated for male and female genders in all postures and positioning to assess bilateral symmetry, and we did not find a difference between the angular values in any of the four positions evaluated for both genders. (Table 2)

**Table 2:** Comparison between right and left sides.

Variables	p values – masculine	p values – feminine
LPF	0.316	0.0515
LAF	0.241	0.2619
OAF	0.124	0.0520
OPF	0.259	0.0712

LPF: lying parallel feet; LAF: lying abducted feet; OAF: orthostatic abducted feet; OPF: orthostatic parallel feet.

Table 3 shows the comparison of the angle Q between the positions of the feet, separating the lying and standing postures. When analyzed separately for each gender, we found significant differences between the values ​​in orthostatic position with open feet and parallel feet on the left side in both gender, as well as for the overall group.

**Table 3:** Comparison between values of Q angle changing feet positioning.

Variables	p values – masculine	p values – feminine	p values –Total Group
LPF L & LAF L	0.507	0.127	0.462
LPF R & LAF R	0.629	0.576	0.985
OAF L & OPF L	0.000*	0.000*	0.000*
OAF R & OPF R	0.126	0.930	0.261

(*) p < 0.05; LPF: lying parallel feet; LFA: lying abducted feet; OAF: orthostatic abducted feet; OPF: orthostatic parallel feet.

Comparing the positions evaluated with each other without crossing between right and left limbs, there was a statistically significant difference only when comparing the orthostatic position with parallel feet and the orthostatic with abducted feet of the left hemi body. (Table 4)

**Table 4:** Comparison between positions for the total group (N = 62).

Variables	p values - Left	p values - Right
LPF X LAF	0.995	0.999
LPF X OAF	0.969	0.246
LPF X OPF	0.089	0.104
LAF X OAF	0.996	0.248
LAF X OPF	0.050	0.106
OAF X OPF	0.027*	0.976

(*) p < 0.05; LPF: lying parallel feet; LFA: lying abducted feet; OAF: orthostatic abducted feet; OPF: orthostatic parallel feet.

## DISCUSSION

The evaluation of the Q angle of 62 university students of both genders was performed, aiming to assess possible variations in measurement. The evaluation was performed following a previously established protocol based on extensive literature review.

The sample comprised university students of both genders in the age group 17-31 years old. In this age group several studies were conducted,^2^^,^ 5 ^-^ 7 ^,^ 13 ^-^ 15 and anterior knee pain symptoms and pathologies such as patellofemoral dysfunction were present. Also at this age, the knee do not present bone growth, and there are no degenerative pathologies that could change the Q angle.

The methodology used in this study comprised the use of sensitive and reliable equipment, using the caliper and computed photogrammetry.^9^^,^ 11 The caliper was suggested by France and Nester^9^ as an indispensable tool for demarcation points of difficult anatomical location, minimizing errors in this item. Another important item was the demarcation and imaging in three takes, allowing a more reliable effect the results found.^9^ Finally, our methodology chose a validated and reliable tool, computed biophotogrammetry, which allows accurate quantitation and is non-invasive.^11^ Studies like Tsujimoto's *et al*.^16^ used computed tomography or radiography, which are reliable but costly resources, and expose the volunteer to radiation. Other assays such as Tomsich *et a*l.^17^ Kuhn *et al*.^4^ used goniometry which has questionable reliability and reproducibility.

Assessment of quadriceps angle is commonly performed unilaterally, since the limbs would be symmetrical. However, Livingston^18^ emphasizes the need for studies with bilateral assessment of limbs. The present study compared the value of bilateral Q angle, which resulted in a statistical symmetry in all positions analyzed. Just as Herrington and Nester,^6^ evaluating 109 young adult volunteers in orthostatic position, parallel feet. Thus, this study confirms the reliability of the current data, as the author herein cited evaluated a larger group and found results similar to this study.

On the other hand,Hahn and Foldspang,^14^ studying 339 athletes found differences between the right and left Q angles. To Livingston and Spaulding,^5^ however, the reason for this difference is unclear and these authors do not suggest any explanation for the fact. For Livingston and Mandingo,^8^ the difference of values between both sides is explained by the higher tropism and muscle tone in the dominant hand, which would cause a force on the patella displacing it and decreasing the value of the angle. For Raveendranath *et al*.,^19^ however, the difference in values between the lower limbs can be attributed to a change in the relative position of the tuberosity of the tibia regarding the center of the patella.

The discrepancy in results between this study and that of Hahn and Foldspang,^14^ should be the use of more precise methodology, by using the average of three measurements, and the use of the caliper to locate the center of the patella. Still on the difference between results, Weiss *et a*l.^20^ reported that goniometric measurements may show up to 3 degrees of discrepancy between raters.

It has been suggested in the literature the use of orthostatic and supine postures^5^^,^ 6 ^,^ 8 ^,^ 14 associating to these external and internal rotation of lower limbs.^5^^,^ 7 ^,^ 21 It is also important in evaluating the Q angle to ascertain the degree of contraction or relaxation of the quadriceps muscle,^5^^,^ 6 ^,^ 8 ^,^ 15 ^-^ 17 because the patella rises when this muscle is contracted.^15^^,^ 17

The adoption of knee extension for evaluation of Q angle was based on studies by Smith *et al*.^22^ whereby there is greater intra-rater reliability when the knee is assessed in full extension compared to 20° and 24° of flexion. Still on the knee positioning, Greene *et al*.^23^ verified the validity of the evaluation criterion Q angle in full extension of the knee compared with 20° knee flexion, and reported that there was a weaker correlation between the findings of the clinical and radiological assessments of the Q angle with the knee in flexion compared to full extension. That said, this study evaluated the Q angle always with the quadriceps muscle relaxed and knee in full extension, since besides being the most used method in present studies,^5^^,^ 6 ^,^ 8 ^,^ 15 ^,^ 16 ^,^ 23 it was of interest to evaluate the afore mentioned angle without changes imposed by muscle contraction.

The current study evaluated 62 asymptomatic volunteers and showed no difference between the angular values ​​by changing the rotation of the lower limbs in the supine position. Olerud and Berg 21 evaluating 34 asymptomatic young adults, with internal and external rotations in the supine position, found that the magnitude of the Q angle increases in internal rotation and decreased in external rotation. This difference in results is attributed to the larger number of volunteers in the present study, by using a more reliable location of anatomical points, and using the average of three values ​​for each position, which provides greater reliability and veracity to the results that have been presented. When comparing the values ​​of the angle in the standing position with the internal and externally rotated lower limb, in both genders, revealed a difference only on the left side.

Livingston and Spaulding^5^ evaluated the Q angle in the standing position in 20 individuals of both genders through computerized analysis in three positions. They found differences in the comparison between all positions for both members. This study found similar results, as was no significant difference between the rotations in the standing posture, for both genders, in the left lower limb. When it refers to the right limb, the dominant side in 95% of the population studied, this difference was not found. This is attributed once again to the fact that we used a more thorough method in that angle evaluation. As suggested by France and Nester,^9^ for more confident results and to reduce evaluation errors, we used precise equipment to locate the center of the patella, and three measurements of the angle were carried out. In addition, this study evaluated a larger number of volunteers, giving greater credibility to these results when compared to the study of Livingston and Spaulding.^5^

On the right lower limb no difference was found between the placement of members with internal and external rotation in the orthostatic position. Among the possible explanations is the possibility that there are changes in muscle electrical activity between dominant and non-dominant members, as suggested by Ounpuu and Winter.^24^ Bagesteiro and Sainburg^25^ showed central difference in motor control, keeping the muscles of dominant lower limbs with increased tone. Changes in muscle electrical activity and torque may be present in the volunteers evaluated with the same request for relaxation of the lower limb muscles, since changes in the alignment, as tibial rotation and anteversion of femoral neck generates co-contractions in the muscles of the knee and hip beyond the control of the volunteer as reported by Bagesteiro and Sainburg.^25^ This, in turn, would alter the position of the limb, causing Q angle alteration. Another possible explanation for the difference that has occurred between the dominant and non-dominant sides is the rotation is femur, as described by Sanfridsson *et al*.,^26^ who reported that the biomechanics of the lower limb varies with the position of the limb and the medial rotation of the femur may lead to an increase in values of the Q angle ​​, and vice versa, or still be related with tibial rotation. However, these variables were not controlled in the present study.

Livingston and Spaulding,^5^ after studying the Q angle in the standing position with different rotations, concluded that the ideal posture is standing with parallel feet. The assessment with external rotation is not recommended because it produces discomfort to the patient and may represent an altered angle. The present work agrees with the above-mentioned regarding the discomfort of maintaining external rotation, once the volunteers reported more discomfort and greater difficulty in keeping quadriceps relaxed in this position. Thus, Livingston and Spaulding^5^ suggest that in future studies orthostatic posture and parallel feet is used. On the other hand, Olerud and Berg^21^ reported that the assessment should be in the supine posture, and reported this to be the best for assessing the Q angle. Observing the variables, we have the conviction that an important point when evaluating the Q angle is to use precise methodology, reducing measurement errors. Regarding the most appropriate posture, supine posture stands out because it has no influence on muscle and femoral rotations imposed by orthostatism.^16^ Regarding the rotation, the internal seems to be the best, to be easily positioned as the positioner is not needed and it can be performed at any location.

This study is limited with regard to the control of the positioning of the femoral neck (anteversion of the femoral neck), since radiographic images were not analyzed in order to measure this variable.

From the results obtained, it appears that there are differences in the value of the Q angle between positions with internal and external rotation of the lower limb in orthostatic position, however, when comparing the Q angle in the supine posture no statistical differences were found, i.e., the values ​​of the Q angle does not change with the limb rotation in this position, suggesting that because of the absence of muscle contraction (promoting muscle relaxation and release of the patellofemoral joint) this position can be adopted to be the most neutral, minimally influencing the positioning of the patella and tibia.

## CONCLUSION

From the data analysis, it can be considered that among the population studied, in the supine position there is no asymmetry of the Q angle independently of hip rotation, which does not happen in the orthostatic position. From the conclusion above, it may be suggested that the Q angle can be measured in the supine position without rotation of limbs, a position easy to adopt and standardize for the measurement of this angle clinically.
